# Smouldering Lesion in MS: Microglia, Lymphocytes and Pathobiochemical Mechanisms

**DOI:** 10.3390/ijms241612631

**Published:** 2023-08-10

**Authors:** Dániel Pukoli, László Vécsei

**Affiliations:** 1 Department of Neurology, Esztergomi Vaszary Kolos Hospital, 2500 Esztergom, Hungary; pukoli.daniel@med.u-szeged.hu; 2 Department of Neurology, Faculty of Medicine, University of Szeged, Semmelweis u. 6., H-6725 Szeged, Hungary; 3 Danube Neuroscience Research Laboratory, ELKH-SZTE Neuroscience Research Group, Eötvös Loránd Research Network, University of Szeged (ELKH-SZTE), Tisza Lajos krt. 113, H-6725 Szeged, Hungary

**Keywords:** glutamate excitotoxicity, lymphocyte, microglia, mitochondrial dysfunction, multiple sclerosis, neuroinflammation, neurodegeneration, oxidative stress, slowly expanding lesion, smouldering lesion

## Abstract

Multiple sclerosis (MS) is an immune-mediated, chronic inflammatory, demyelinating, and neurodegenerative disease of the central nervous system (CNS). Immune cell infiltration can lead to permanent activation of macrophages and microglia in the parenchyma, resulting in demyelination and neurodegeneration. Thus, neurodegeneration that begins with acute lymphocytic inflammation may progress to chronic inflammation. This chronic inflammation is thought to underlie the development of so-called smouldering lesions. These lesions evolve from acute inflammatory lesions and are associated with continuous low-grade demyelination and neurodegeneration over many years. Their presence is associated with poor disease prognosis and promotes the transition to progressive MS, which may later manifest clinically as progressive MS when neurodegeneration exceeds the upper limit of functional compensation. In smouldering lesions, in the presence of only moderate inflammatory activity, a toxic environment is clearly identifiable and contributes to the progressive degeneration of neurons, axons, and oligodendrocytes and, thus, to clinical disease progression. In addition to the cells of the immune system, the development of oxidative stress in MS lesions, mitochondrial damage, and hypoxia caused by the resulting energy deficit and iron accumulation are thought to play a role in this process. In addition to classical immune mediators, this chronic toxic environment contains high concentrations of oxidants and iron ions, as well as the excitatory neurotransmitter glutamate. In this review, we will discuss how these pathobiochemical markers and mechanisms, alone or in combination, lead to neuronal, axonal, and glial cell death and ultimately to the process of neuroinflammation and neurodegeneration, and then discuss the concepts and conclusions that emerge from these findings. Understanding the role of these pathobiochemical markers would be important to gain a better insight into the relationship between the clinical classification and the pathomechanism of MS.

## 1. Introduction

Multiple sclerosis (MS) is a chronic disease characterised by inflammation, extensive primary demyelination and progressive neurodegenerative processes [1]. Although a wealth of interesting information on the pathogenesis of MS has been accumulated over the past decades, the exact cause of this disease remains to be elucidated. In addition to environmental and genetic factors, much interest has focused on the role of various infectious agents. In particular, the role of the herpes virus family, *Epstein-Barr virus (EBV)* and *human herpesvirus 6 (HHV-6A)* has been the subject of much research interest. A recently published study suggests that age-dependent *EBV* infection may contribute to the development of MS, while based on the presence of HHV-6A antibodies suggests that the development of MS is independent of age [2].

In everyday medical practice, MS has two main clinical presentations: relapsing-remitting MS (RRMS) and progressive form (PMS). The first clinical presentation of MS is a clinically isolated syndrome (CIS), which falls within the spectrum of RRMS. The progressive form includes primary progressive multiple sclerosis (PPMS) and secondary progressive multiple sclerosis (SPMS) [3]. A better understanding of the pathomechanisms of RRMS and SPMS and, just as important, a better understanding of the molecular mechanism of the transition from RRMS to SPMS are some of the major challenges in MS research. Recently published results have confirmed that long-term disability in MS is largely independent of relapses (progression independent of relapse activity, PIRA) and correlates well with brain atrophy detected on MRI images. Particularly relevant in this regard is the study by Cree et al., which included 138 patients. In this group, 92 patients diagnosed with RRMS had evidence of silent progression, which could certainly be related to the neurodegenerative process in RRMS [4]. This silent progression is likely to be present in many patients diagnosed with RRMS. The same factors are likely to be responsible for the clinical symptoms of SPMS when clinical worsening is clearly evident. Therefore, the pathophysiological events in SPMS likely occur much earlier than is currently thought. A better understanding of this process also has important implications for therapeutic decision-making.

Significant progress has been made in recent years in understanding the pathomechanism of MS. However, little is known about the underlying cellular and molecular mechanisms that influence lesion formation and progression in individual patients. Understanding the role of these pathobiochemical markers would be important, as they may, in many cases, have a major impact on correct therapeutic decisions and gain a better insight into the relationship between the clinical classification and the pathomechanism of MS. In this review, we will discuss how these pathobiochemical markers and mechanisms, alone or in combination, lead to neuronal, axonal and glial cell death and, ultimately, to the process of neuroinflammation and neurodegeneration. We will also discuss what concepts and conclusions can be drawn from these findings.

## 2. MS Lesion Pathology

**Figure 1:**
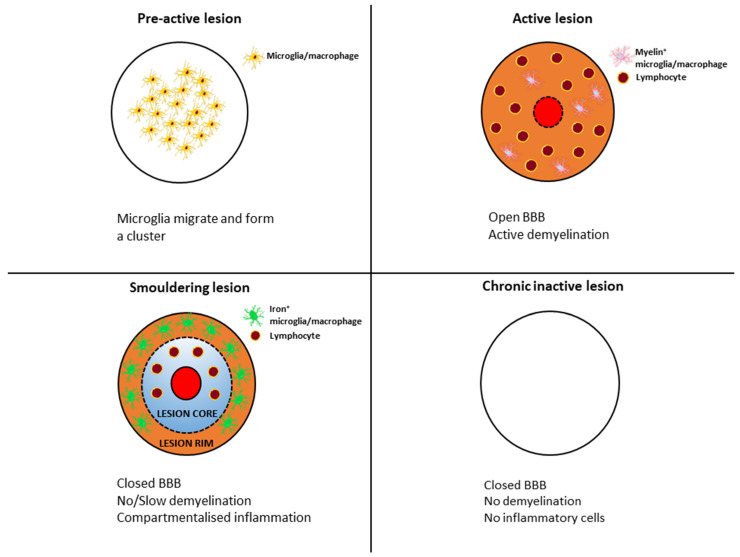
Transformation of typical lesion patterns in the course of multiple sclerosis.

The pathological hallmarks of MS lesions are inflammation, demyelination, axonal damage and gliosis. The pathological process involves the formation of lesions in the central nervous system (CNS) consisting of lymphocytes, macrophages and glial cells, leading to demyelination and axonal loss. MS plaques form in the brain and spinal cord, mainly, but not exclusively, in the white matter around the ventricles, the optic nerves and tracts, the corpus callosum, the cerebellar peduncles, the long tracts, and the spinal cord and brainstem. They are present in all forms of MS, although their quantities and composition vary between the different forms of MS and as the disease progresses. Different types of lesions (see Figure 1) can be distinguished at different stages of MS based on their degree of microglial activation, inflammatory and demyelinating activity [5]. According to neuropathological data, pre-active lesions are characterised by clusters of activated microglia in areas of otherwise normal-appearing myelin [6]. Active MS lesions show infiltration of lymphocytes around a central vein, accompanied by microglial activation and the presence of macrophages attracted to the lesion, which produce pro-inflammatory cytokines and inflammatory mediators. Therefore, are thought to be involved in the development of chronic axonal loss. B-cells and plasma cells remain mainly in the perivascular space, while CD8+ T-cells diffusely infiltrate the lesion parenchyma [7,8]. Macrophages and microglia contain myelin degradation products, indicating that they facilitate the degradation of myelin proteins [9,10]. These lesions are most common in early MS (acute and RRMS) but become rare during progressive MS [11], and there is marked inflammation, significant damage to the blood–brain barrier (BBB) and concomitant diffuse active demyelination in the lesion associated with massive microglial infiltration [1]. However, with the impenetrable BBB in the CNS, compartmentalised inflammation may persist as chronic inflammation. Most acute inflammatory lesions in MS subsequently become inactive (chronic inactive lesions) and shrink due to gliosis. This chronic inflammation is thought to underlie the development of so-called chronic active lesions (also commonly referred to as “mixed active–inactive” or “smouldering” lesions) [7,11,12]. Smouldering lesions show a low-grade chronic inflammation characterised by chronic axonal damage and concurrent demyelination and are further characterised by a gradual increase in size towards the normal-appearing white matter (NAWM) [11,13]. Based on autopsies, 20–40% of white matter lesions are such lesions, with CD8+ T- and CD20+ B lymphocytes in the centre with a small number of plasma cells, surrounded by a loose network of iron-loaded activated microglia cells and macrophages and proliferating oligodendrocyte cells at the periphery [8,14].

**Figure 2:**
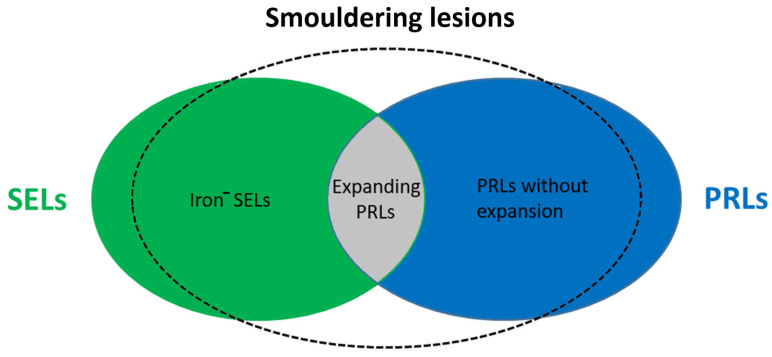
Representation of smouldering lesions in MS. Iron^−^—iron negative; SEL—slowly expanding lesion; PRL—paramagnetic rim lesion.

These lesions have previously only been properly studied in histological specimens obtained from autopsies but can now be detected by MRI (7-Tesla, 3 T and 1.5 T) [15,16,17]. Slowly expanding lesions (SELs) represent a subgroup of chronic white matter lesions in MS that show gradual expansion over time and have emerged as a potential in vivo marker of smouldering lesions detectable on longitudinal T1-weighted and T2-weighted MRI [18,19]. In chronic lesions, iron-laden microglia and/or macrophages form a paramagnetic rim (paramagnetic rim lesions, PRLs), which can be visualised by susceptibility-weighted imaging (SWI) MRI and are characterised by more severe tissue damage [16,20,21]. Recent evidence suggests that SELs only partially align with PRLs, suggesting that SELs, whether with or without phase rims, and PRLs, with or without slow expansion, may represent different aspects or stages of MS pathology within smouldering lesions (see Figure 2) [22]. A longitudinal clinical study evaluating more than ten years of MRI reports found a strong association between the number of rim-positive chronic lesions (RPCL, a hypointense rim on phase images and internal isointensity to extralesional white matter) and cognitive impairment and the risk of clinical progressive MS, but further studies are needed to assess the negative prognostic significance of chronic lesions [12]. PRLs have been observed in patients with relapsing and progressive disease and may have prognostic value for long-term disability. It has been suggested that PRLs may be a biomarker for smouldering lesions and compartmental inflammation in MS [12,17,23]. During the course of the disease, the proportion of smouldering lesions increases over time and is higher in progressive than in relapsing-remitting disease and is rare in RRMS [11]. In addition, these lesions have also been shown to correlate with disability and predict progression in both relapsing-remitting and secondary progressive MS [19,24]. This suggests that the presence of smouldering lesions increases the risk of transition to progressive MS and may also be an anatomopathological feature of PIRA or silent progression, i.e., progression in the absence of clinical and radiological signs of inflammatory activity, which is a characteristic feature of progressive MS and can be detected in the early course of the disease [4].

In summary, smouldering lesions evolve from acute inflammatory lesions and are associated with continuous, low-grade demyelination and neurodegeneration over many years. Their presence is associated with a poor disease prognosis and promotes the transition to the progressive stage, which may later manifest clinically as progressive MS when neurodegeneration exceeds the upper limit of functional compensation.

## 3. Immunopathology of MS

Multiple sclerosis is a chronic immune-mediated inflammatory and neurodegenerative disease of the central nervous system. The pathogenesis of MS is not fully understood despite decades of intensive research. The widely accepted concept of its pathogenesis is that T-cell-mediated inflammation in the brain and spinal cord initiates tissue damage. Then, demyelination and neurodegenerative lesions are driven by heterogeneous mechanisms involving both adaptive and innate immune systems [1,25].

### 3.1. T-Cells in the Immunopathology of MS

Initially, the inflammatory response observed in MS was thought to be mediated by MHC class II antigen-restricted CD4+ T lymphocytes, and this was supported by a large body of experimental and clinical data. However, recent neuropathological data and experience from clinical trials do not support a dominant role for these cells in the progression of established multiple sclerosis. This finding is supported by the predominance of CD8+ T-cells over CD4+ T-cells among T-cells infiltrating acute and chronic MS lesions and by the fact that drugs that specifically block CD4+ T-cells have been shown to be ineffective. Therefore, CD4+ T-cells are thought to contribute to the immune response initiation in MS patients but less in the effector stage of inflammation, immune-mediated demyelination and neurodegeneration [8,26]. These developments have focused attention on the possible role of other immune cells in the pathophysiology of MS. Research published in recent years has shown that CD8+ cytotoxic T-cells, B-cells and antigen-presenting cells (APCs) may be more important in the presentation and spreading stages of brain and spinal cord lesions.

In cortical lesions of MS patients, CD8+ T-cells with a phenotype corresponding to tissue-resident memory cells associated with disease progression, meningeal inflammation and neurodegeneration [27,28] have been found to proliferate in the brain in a focal manner (detected in high numbers and early in MS plaques) and show signs of activation [8,29] or clonal expansion [30]. They may have a dual role: first, they recognise antigens presented by MHC I molecules on the surface of axons and myelin-producing glial cells and, through their cytotoxic function, can damage these cells and the BBB, thus acting as effector cells for demyelination and axonal destruction, thereby promoting disease development; secondly, they are also able to inactivate CD4+ T-cells and inhibit their proliferation [31,32]. These data suggest that CD8+ T-cells either promote disease development or play a regulatory role.

The recognition of Th17 and Treg cells is a major discovery in immunological research. When naïve cells, i.e., cells that have not yet encountered antigen, are exposed to various cytokine effects, they can differentiate into Th1, Th2 or Th17 lineages. Th17 cells produce IL-17 (hence their name) and the cytokine IL-22, which enhance neutrophil migration and endothelial permeability via the IL-17 receptors (see review [33]). IL-17 is also involved in the activation of microglia, macrophages and astrocytes. In MS patients, interferon-β treatment inhibits Th17 generation resulting in decreased IL-17 levels [34,35]. While Th17 is an important cell lineage that propagates autoimmunity, CD4+CD25+FoxP3+ Treg cells inhibit pathological autoimmunity. Treg cells, like other T-cells, mature in the thymus and are thought to arise from T-cells that are highly reactive to their own antigens expressed in the thymus and have an important function in inhibiting the activation of autoimmune T-cells (see review [36]). Treg cells are potential therapeutic targets in autoimmune diseases; several other molecules already in clinical trials affect their function [37,38,39,40]. Exciting results on mRNA therapy for experimental encephalomyelitis (EAE) have recently been published [41]. After administration of the specific mRNA, the number of T effector cells was reduced, and the treatment also had a beneficial effect on the regulatory T-cell population. The regulatory T-cells had a significant immunosuppressive effect. These data suggest a novel mRNA-based gene therapy approach for the treatment of MS.

### 3.2. B-Cells in the Immunopathology of MS

The success of B-cells-based therapies has shed new light on our current concept of the pathomechanism of multiple sclerosis. As early as the 1940s, research showed that immunoglobulin concentrations were increased in the cerebrospinal fluid (CSF) of MS patients, and the role of B-cells in the pathomechanism of MS became an area of great interest in neuroimmunology. Detection of oligoclonal IgG bands (OCB) has been demonstrated in a large proportion of patients. Mature plasma cells derived from B-cells produce antibodies and induce OCB, which are present in at least 90–95% of MS patients [42]. Antigen-specific memory B-cells are also present in the CNS of patients and can be detected in MS lesions [43]. This, together with the presence of antibody and complement deposition in MS lesions and evidence of antigen-induced clonal expansion of B-cells in the CNS [44], suggests that B-cells may contribute to MS pathology by producing autoantibodies specific to CNS antigens [42]. However, independent of their antibody-producing activity, B-cells can also be involved in temporary or permanent damage to nerve tissue. Indeed, they are able to present antigens to T-cells and release pro-inflammatory (IL-6, IL-12, IL-15, TNF-alfa, IFN-gamma, granulocyte-macrophage colony-stimulating factor [GM-CSF]) and anti-inflammatory cytokines that regulate immune processes [45]. An abnormal B-cell cytokine balance between inflammatory and pro-inflammatory cytokines and antigen-presenting capacity has been observed in the peripheral blood of MS patients [42]. This aberrant cytokine profile is thought to contribute to the abnormal T-cell response in MS and may underlie the therapeutic effect of B-cell depletion [46]. In addition, the presence of follicle-like structures (FLS) described in B-cell-rich meninges suggests that they may play an important role in the progressive phase of the disease. In MS, B-cell-rich FLS were detected in the meninges (more frequently in SPMS) based on subpial cortical damage [47,48,49]. The presence of such FLS is associated with an earlier onset and a more progressive course of the disease. The extent of cortical neuronal loss also suggests that soluble factors released from these structures play a pathogenic role in the progressive phase of MS [47]. An in vitro study by Lisak et al. has shown that B-cells exert a cytotoxic effect on in vitro oligodendroglial cultures, which may be a factor in demyelination [50]. However, it should be noted that B-cell-derived immunoglobulins do not necessarily react with the myelin protein in brain tissue [42,51]. Therefore, the pathogenicity of immunoglobulins produced in the CNS of MS patients remains uncertain. Thus, B-cells may contribute to the pathogenesis of MS and neuronal tissue damage through multiple mechanisms, including (1) antibody production, (2) antigen presentation, (3) modulation of the T-cell response, (4) activation and proliferation of auto-proliferative CD4+ T-cells in the CNS, (5) pro-inflammatory cytokine and chemokine production, and (6) compartmentalisation of pathological processes. It can be concluded that B-cells play an important role in the development and progression of multiple sclerosis through various antibody-dependent and -independent effects.

Research studies published in recent years have demonstrated that B lymphocytes play a crucial role in the pathogenesis of MS. Therefore B-cell depletion therapies are becoming increasingly crucial in reducing disease progression. Another potential target for inhibiting B-cell function is the B-cell maturation cytokine BAFF (B-cell Activating Factor) and A Proliferation-Inducing Ligand (APRIL) [52].

### 3.3. Microglia in the Immunopathology of MS

Microglia cells are the primary immune cells of the central nervous system that originate from yolk sac progenitor cells early in embryogenesis and continue to exist throughout life [53,54,55]. They have a remarkable ability to adapt and differentiate based on the inflammatory conditions present during various stages of diseases, including CNS injuries, neurodegeneration, and infections. They maintain homeostasis in the CNS and can stimulate neuroprotective processes or activate neurotoxic pathways depending on their specific phenotype [56]. These phenotypes are characterised by the presence of specific cell surface molecules and the production of specific cytokines and chemokines. M1 microglia can trigger neurotoxic pathways, promote inflammation, damage oligodendrocytes, and produce pro-inflammatory mediators such as NO (nitric oxide), ROS (reactive oxygen species), IL-1β, and TNF-α leading to progressive neurodegeneration. On the other hand, the M2 phenotype, classified into M2a, M2b, and M2c subtypes, is associated with reparative and immunoregulatory functions, promoting phagocytosis and producing various factors like Arg1 (arginase 1), CD206, IGF-2 (insulin-like growth factor), and anti-inflammatory cytokines such as IL-10 [57,58,59,60].

#### 3.3.1. Neurotoxic Microglia

In the context of multiple sclerosis, neuropathological studies have highlighted the role of chronically activated microglia in disease progression. Patients with progressive MS showed either chronic active (smouldering or expanding) lesions with microglial activation at the edge of the burned plaque or inactive lesions without microglial activity [11]. In patients with progressive MS, single-cell mass cytometry analysis has shown that highly phagocytic and activated microglia downregulate the expression of homeostatic markers (such as P2Y12 and GPR56) and upregulate the expression of proteins involved in microglial activation and phagocytic activity including CD68, CCR2, CD64, CD32, CD95, and CCL4 [61]. Microglia can produce a range of pro-inflammatory cytokines (IL-6, IL-1β, C1q and TNF-α) and chemokines (CCL2, CCL3, CCL4 and CCL5) during CNS inflammation in both MS and its animal model, EAE. They may induce deleterious effects (such as tissue damage, demyelination, and cell death) on neighbouring neurons and glial cells. [62]. Microglial TNF-α and C1q have been implicated in the induction of a neurotoxic A1 astrocyte phenotype, causing rapid death of oligodendrocytes and neurons [63,64]. Unlike typical astrocytes, which support neuronal survival, synapses and the integrity of the BBB, these reactive astrocytes reduce their supportive role and begin to produce neurotoxic factors, complement components such as C3 and chemokines (such as CXCL10). These may help to attract immune cells across the BBB into the CNS [65]. New research shows that an interaction between immune cells and glial cells in the brain, mediated by the complement system protein C1q, appears to drive chronic inflammation in MS [66]. These findings suggest that pharmacological inhibition of C1q may be a potential new therapeutic approach to address chronic inflammation in MS. Microglia also play a role in mitochondrial damage associated with axonal damage, enhancing mitochondrial injury by producing reactive oxygen and nitrogen species [67,68]. They also take up and release iron from damaged oligodendrocytes, potentially contributing to oxidative stress and further neuronal and axonal destruction [69]. It has been hypothesised that activated microglia in smouldering lesions play a role in inhibiting remyelination and that the released inflammatory mediators further promote the development of axonal damage through oxidative stress. A phase III clinical trial is currently investigating the therapeutic efficacy of Bruton’s tyrosine kinase (BTK) inhibitors in MS; these molecules inhibit microglial activation and are thought to promote a switch in microglial phenotype from a pro-inflammatory to a pro-myelinating type [14]. One potential criticism of the short follow-up period used in this study may be in connection with the effect of BTK inhibitors on chronic inflammatory phenotypes (e.g., smouldering lesions).

In the early phase of the disease, microglial activation promotes the recruitment of naive T-cells, which are then activated by dendritic cells assuming the role of antigen-presenting cells. In several studies, microglia-expressing markers associated with T-cell co-stimulation have been detected in actively demyelinating lesions. Microglia also act as antigen-presenting cells, leading to the re-stimulation of autoreactive memory T-cells entering the CNS through the BBB, which is likely to be a key event in the maintenance of chronic CNS inflammation [70,71]. Activated microglia and astrocytes may contribute to the persistence of B-cells within the CNS by secreting molecules, such as BAFF and interleukin (IL)-6, which are known to support B-cell survival [72]. Active white matter lesions show a widespread intensive infiltration of activated microglia and macrophages. In contrast, smouldering lesions are surrounded by a dense rim of concentrated pro-inflammatory markers (such as CD68 and p22phox) expressed by microglia and macrophages [9,14,73]. Microglial activation is not restricted to lesions but is also diffusely present in non-lesional white and grey matter with concomitant axonal degeneration and meningeal inflammation [74].

#### 3.3.2. Available Biomarkers of Microglia

TSPO (translocator protein) is a mitochondrial molecule upregulated during microglial activation. TSPO PET scans have shown significant diffuse microglial activity in progressive MS, independent of focal lesions, which may be related to the neurodegeneration process itself. In normal-appearing white matter, microglial activity is very active around focal lesions, and high microglial activity is also observed around so-called burned-out plaques. However, for a more nuanced assessment, it is important to note that TSPO is not a specific microglial marker, as it is expressed on vascular endothelium as well as on activated astrocytes and does not distinguish between pro- and anti-inflammatory microglia. Recently, several other microglial markers have been developed for PET scans, including markers for iNOS (inducible nitric oxide synthase), IDO-1 (indoleamine 2,3-dioxygenase-1), KMO (kynurenine-3-monooxygenase) and CB2 (cannabinoid receptor 2) detection [75].

#### 3.3.3. Neuroprotective Microglia

Microglia can also have a neuroprotective function. There are several key mechanisms by which microglia contribute to neural repair and remyelination, including the clearance of myelin debris via phagocytosis and the secretion of anti-inflammatory cytokines such as IL-4, IL-10, and IL-13 [76,77]. Several receptors, including toll-like receptor (TLR), TREM2, CRs, FC, and PSR, have been implicated in the phagocytic function of microglia. For instance, TREM2-deficient microglia show an impaired upregulation of genes associated with phagocytosis and lipid metabolism [78,79]. In addition to clearing debris, microglia contribute to remyelination by promoting the recruitment of oligodendrocyte precursor cells (OPCs) to the lesion site and fostering their proliferation and differentiation. This is achieved in part by switching from a pro-inflammatory (M1) to a neuroprotective (M2) microglial phenotype, as observed in models of lysophosphatidylcholine (LPC)-mediated demyelination [80,81,82]. Alternative activated microglia (M2a) are induced by anti-inflammatory cytokines (such as IL-4 and IL-13), and play a role in repair and regeneration processes. They are also involved in collagen formation and tissue repair. Transitional activated microglia (M2b) are associated with immune regulation. These cells are generated when stimulated by immune complexes in the presence of a TLR ligand. They express high levels of anti-inflammatory IL-10 and low levels of IL-2 and also upregulate MHC-II and CD86. They may also facilitate the recruitment of regulatory T-cells. Acquired deactivating phenotype (M2c) is associated with tissue repair and immunoregulatory functions. These cells are potently induced by IL-10, TGF-β, or glucocorticoids and predominantly produce high levels of IL-10 and CD163 but low levels of Th1 cytokines [57,83,84,85]. Despite the neuroprotective potential of microglia, failure of remyelination and progressive demyelination are considered central to disease progression in MS. Reduced numbers of OPCs within lesions have been observed in patients with progressive disease [80,81], suggesting a potential failure of OPC recruitment. The exact mechanism underlying the transition from pro-inflammatory to pro-regenerative microglia remains elusive, but microglia cell death by necroptosis has been proposed as a possible mechanism [86].

The role of activated microglia in MS and other neuroinflammatory and neurodegenerative diseases remains unclear. While some suggest that these cells help to maintain the damaged CNS, for example, by phagocytosing debris and limiting inflammation, others suggest that overactive microglia interacting with injured neurons could perpetuate a self-perpetuating cycle of prolonged inflammation, thereby driving chronic disease progression [87]. However, it is well established that activated microglia are present in the MS-affected brain, regardless of whether they are causing or merely observing the disease process. Accordingly, chronic microglial activation has been associated with neurodegeneration in the progressive phase of MS [48,74,88]. PET scans can also be used to differentiate between chronic active and inactive lesions. It has been mentioned earlier that smouldering lesions are a major contributor to MS progression. Nowadays, it is possible to detect these lesions in patients and thus study the kinetics of plaque evolution. These data could contribute significantly to a more accurate assessment of disease progression. It may become possible to perform longitudinal in vivo follow-up of the pathobiological events underlying progressive MS. Overall, these findings underscore the intricate role of microglia in MS and highlight the need for further studies to fully understand their complex functions and potential as therapeutic targets.

## 4. Pathogenetic Implications in MS

Despite the traditional view of MS as a two-stage disease with a transition from an inflammatory to a neurodegenerative stage, recent pathological studies have shown that active inflammation and demyelination persist in the CNS even in the late stages of MS [14]. Whether the inflammatory process in MS is the primary driver of tissue damage or whether lesion formation is triggered by a neurodegenerative process that is enhanced or modified by the inflammatory response in MS remains controversial. Genetic studies and experience in animal models suggest that inflammation precedes neurodegeneration [89]. However, from a biological point of view, this is a process between relapsing and progressive MS, with mainly quantitative rather than qualitative differences in pathology.

### 4.1. Neuroinflammation in MS

**Figure 3:**
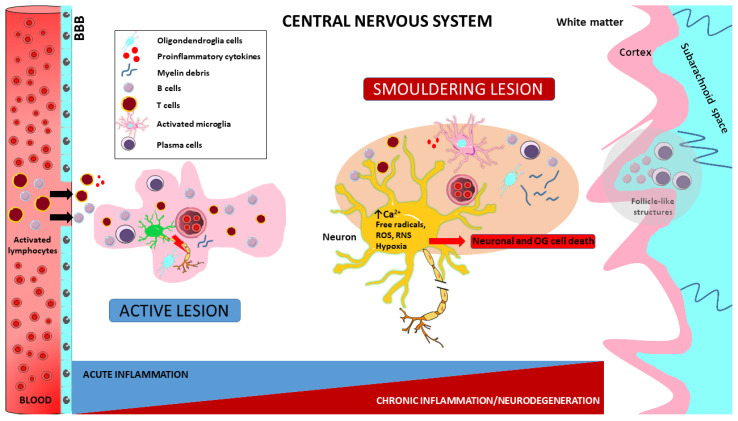
Pathogenetic implications in MS. Impaired autoreactive T-cell function is a consequence of Foxp3 T-cell dysregulation leading to T-cell maturation and proliferation induced by unidentified extrinsic or intrinsic antigens. The resulting activated T lymphocytes engage antigen-presenting B lymphocytes and induce B-cell maturation and differentiation into antibody-secreting plasma cells. Both B and T lymphocytes cross the blood–brain barrier (BBB) and enter the CNS. This results in damage to myelin, oligodendrocytes and prephagocytic neurons and the release of highly pro-inflammatory molecules. These, in turn, facilitate the migration of other monocytes and macrophages from the periphery to initiate phagocytosis. In the brain, meningeal B lymphocytes enhance inflammation through antigen presentation and antibody production, leading to cortical demyelination. Macrophages and microglia, which play a fundamental role in innate immunity, are also essential for disease progression by multiple mechanisms. Over the course of the disease, active lesions can evolve into smouldering lesions characterised by compartmentalised inflammation. These lesions consist of a thin rim of activated microglia interspersed with iron and myelin debris, with macrophages/microglia almost completely absent from their centres. In addition, meningeal follicles rich in B-cells are responsible for the subpial cortical demyelination and neuronal degeneration observed in both early and progressive forms of MS. Furthermore, progressive disease and increased neuronal transaction are associated with widespread and even overrepresented activated microglia or microglial nodules, which are already present in early non-lesional MS. Finally, the immune cells involved in these processes release a variety of neurotoxic substances, including antibodies, cytokines, free radicals and proteases. Activated macrophages and microglia release reactive oxygen species (ROS) and reactive nitrogen species (RNS), leading to mitochondrial damage, hypoxia with oligodendroglia (OG) and neuronal cell death.

The underlying pathological picture of MS is highly complex. There is increasing evidence that two different types of inflammation occur in parallel in MS, which are partly independent of each other (see Figure 3). These processes are essential for the development of MS lesions, such as active lesions and smouldering lesions. Acute inflammation is dominated by the peripheral immune response. Auto-reactive T-cells are generated in the peripheral lymphoid tissues and produce pro-inflammatory cytokines that damage the BBB, allowing pathogenic lymphocytes to cross it and enter the CNS. In addition to T-cells, pro-inflammatory cytokines secreted by activated B-cells promote the recruitment and further activation of inflammatory cells. The predominant inflammatory cells are CD8+ T-cells, which proliferate and activate in the early stages of classic active lesions [1,8,26]. The exact mechanism leading to the activation of CD8+ T-cells is still unclear. However, an imbalance between pro- and anti-inflammatory Treg cell phenotypes in the periphery and impaired function of antigen-presenting cells are hypothesised [90]. Within the CNS, T-cells secrete additional pro-inflammatory cytokines and, by recruiting additional immune cells, initiate an inflammatory cascade that mainly involves the formation of new focal lesions in the white matter and also promotes damage to myelin and oligodendrocytes, ultimately leading to focal inflammation, neuroaxonal damage and gliosis [7]. These classic active focal white matter lesions are most common in relapsing-remitting MS and become rare in patients who have entered the progressive stage [11]. Focal inflammation leads to axonal transection, demyelination and microglial activation, and macrophages appear in the area of the inflammatory lesions. Axonal transection can lead to neuronal death through secondary axonal degeneration; demyelinated axons become less conductive and more susceptible to the damaging effects of the pro-inflammatory microenvironment of MS lesions, which can also lead to axonal death [7]. Activation of lymphocytes leads to the activation of microglia and macrophages [48,91]. As mentioned earlier, these cells can adopt an anti-inflammatory phenotype to promote remyelination or become effector cells for myelin destruction and clearance through their pro-inflammatory function. Microglial activation may ultimately lead to the development of focal primary demyelinated areas of the acute inflammatory process with varying degrees of axonal injury [92].

Inflammatory infiltrates formed in the CNS parenchyma and perivascular spaces during the acute phase can have markedly different outcomes: (1) they are cleared by the tissues without resulting in reactive axonal damage or reactive gliosis, allowing recovery and remyelination of the affected area or (2) they can form chronic aggregates that may resemble tertiary lymphoid organs composed of CD4+ and CD8+ T-cells, B-cells and plasma cells, leading to compartmentalised inflammation [47,49].

In the progressive phase of the disease, inflammation (so-called chronic or smouldering inflammation) is present within the CNS behind the intact BBB, and this compartmentalised inflammation drives the neurodegenerative processes responsible for the progressive deterioration of the patient’s condition. This compartmentalised inflammatory response is diffusely present in the meningeal [93] and perivascular Virchow–Robin spaces [94] but may form focal aggregates or structures resembling tertiary lymphoid organs with clearly distinct T-cell, B-cell and plasma cell areas. These follicle-like structures are found in approximately 40% of people with SPMS but are rare in PPMS [93]. Meningeal and perivascular infiltrates are associated with the slow spread of previous focal white matter lesions, diffuse damage to normal-appearing white and grey matter, and subpial cortical demyelination that typically occurs in the brain and spinal cord in the progressive stage of the disease, accompanied by active demyelination and neurodegeneration [48,95,96,97,98]. The perivascular and meningeal inflammatory aggregates associated with lesion activity contain numerous CD20+ B-cells at all stages of the disease (they are most abundant in active lesions), but these appear to transform into plasma blasts and plasma cells as the lesion matures in chronic lesions [8,9,99]. Neuropathological studies have shown that in MS patients, demyelination with microglial activation and axonal damage is observed in the slowly expanding white matter lesions and the cortical and deep grey matter, whereas lymphocytes are located in the meninges [96]. The topographical relationship with the meninges suggests that inflammation may begin in the subarachnoid space surrounded by the meninges, and this may initiate demyelination in all adjacent tissues, both in the cortex and in the white matter of the spinal cord, and then continue to spread deeper into the surrounding tissues [27]. The above data suggest that demyelination and neurodegeneration occur at a distance from T and B lymphocytes and involve the presence of activated microglia and macrophages. Therefore, microglial activation is likely to be mediated by T or B-cell-derived soluble factors. To date, neither the molecular characteristics of the soluble factor nor the antigenic specificity of the infiltrating T- and B-cells have been identified. These data suggest that, on the one hand, the soluble factor(s) produced by inflammatory cells may directly or indirectly cause tissue damage by activating microglia or macrophages and, on the other hand, depending on the stage of the lesion, lymphocytes may play a role in inducing tissue damage or may also play a regulatory role. Microglial activation and production of reactive oxygen and nitrogen species are key mechanisms for the progression of chronic inflammation, demyelination and neuroaxonal degeneration, especially in the progressive stage of the disease [100]. It is important to note that these activated microglia or macrophages are not exclusively confined to active MS lesions. They are also found diffusely distributed within the normal-appearing white and grey matter of the MS brain, particularly in progressive multiple sclerosis [74,101]. Significant accumulations of activated microglia/macrophages associated with amyloid precursor protein (APP)-positive degenerating neurons can be observed in the periphery of lesion areas [102,103]. This finding strongly suggests a role for these cells in PMS and neurodegeneration. Therefore, in addition to classical immune system mediators, it appears that tissue injury may be mediated, at least in part, by a cascade of microglial and macrophage activation leading to oxidative injury and mitochondrial damage, which will be discussed in the following chapters.

Taken together, these data suggest that demyelination and neurodegeneration in MS are driven by the inflammatory process at all stages of the disease, regardless of the clinical phenotype. Several mechanisms of tissue injury have been proposed in relation to MS lesions, such as the role of cytotoxic T-cells, B-cells or microglia and macrophage activation. It is likely that each contributes to some extent to tissue damage in different lesions and lesion stages. Inflammation in the brain and spinal cord is always present in active disease, in the form of classic active lesions in the early stages, smouldering lesions and neurodegeneration in the progressive stages of the disease (see Figure 3). Understanding these mechanisms has influenced our approach to MS and continues to modify it as new scientific advances are made.

### 4.2. Oxidative Stress

The inflammatory response is characterised by the release of a number of inflammatory mediators that can cause tissue damage. Some of these mediators include ROS/RNS, which have been found to play a direct effect in damaging the CNS during MS. ROS are small oxygen-derived molecules that are oxidising agents or can be converted to oxygen radicals [104]. ROS exist in many different forms, with superoxide (O_2_−) and its derivatives hydroxyl radical (OH•) and hydrogen peroxide (H_2_O_2_) being the most abundant. The reaction between superoxide and NO can give rise to peroxynitrite (ONOO-), which is highly reactive and toxic to cells [105]. NO is a reactive molecule that gives rise to several other RNS, including nitroxyl ion (NO-), nitrous acid (HNO_2_), nitrogen dioxide (NO_2_), peroxynitrite (ONOO-), and peroxynitrous acid (ONOOH) [106]. Both activated microglia and infiltrated macrophages are able to generate vast amounts of pro-inflammatory mediators and oxidising radicals, such as superoxide, hydrogen peroxide, hydroxyl radicals and NO [107]. ROS can either damage mitochondrial constituents directly, oxidising mitochondrial DNA and lipids, or form peroxynitrite by reacting with NO, ultimately causing cell death via apoptosis or necrosis [108]. NO and peroxynitrite damage cellular components, inhibit the mitochondrial respiratory chain and cause DNA damage through the overactivation of poly(ADP-ribose) polymerase (PARP) enzymes, causing free radical accumulation, which in turn ultimately leads to cell death through NAD+ depletion [109]. In addition, peroxynitrite can also induce mitochondrial Ca^2+^ efflux by oxidising the thiols of mitochondrial membrane proteins, which can finally lead to the mitochondrial permeability transition. Mitochondrial depolarisation and induction of the permeability transition lead to the release of increased amounts of Ca^2+^ into the cytosol. Increased cytosolic calcium levels caused by mitochondrial damage can lead to cell death by both apoptotic and necrotic pathways. [110]. In addition, ROS/RNS are involved in the maintenance of chronic neuroinflammation, damaging the BBB and thereby promoting the migration of immune cells into the CNS and the secretion of pro-inflammatory cytokines [111].

The production of ROS/RNS is balanced by the scavenging antioxidant system [105]. Important markers of this process are antioxidant molecules that can be measured in the blood (glutathione, alpha-tocopherol, retinol, plasma sulfhydryl groups), as well as uric acid. Studies have confirmed that in MS, blood levels of reducing components change significantly during relapses, in line with increased free radical concentrations [112,113]. Excessive free radical formation and/or a reduction in antioxidant capacity can upset the balance between pro- and antioxidants, leading to oxidative stress [105].

The role of oxidative stress in MS has been demonstrated in recent years in EAE, where increased levels of carbonyl derivatives have been found in various parts of the CNS [114]. In addition, significant levels of nitrotyrosine, a known marker of peroxynitrite-induced degeneration, were detected [115]. Interestingly, even in chronic lesions with fewer immune cells, there is significant evidence of oxidative damage, suggesting an oxidative amplification mechanism within the nervous system. Three major ROS-generating enzymes have been identified in activated microglia and macrophages in MS: myeloperoxidase, xanthine oxidase, and nicotinamide adenine dinucleotide phosphate (NADPH) oxidase. This suggests that initial oxidative damage is driven by the inflammation-induced oxidative burst in activated microglia and macrophages [67]. Microglial activation, production of reactive oxygen and nitrogen radicals, and oxidative damage are key mechanisms for the progression of chronic inflammation, demyelination and neuroaxonal degeneration, especially in the progressive stage of the disease. Oxidative damage can trigger mitochondrial dysfunction and consequent energy deficits, a process known as histotoxic or “virtual” hypoxia. This process is amplified by true hypoxia resulting from increased energy expenditure around inflamed blood vessels. This may explain the accumulation of lesions in so-called watershed areas in the MS brain [116]. Oxidative processes and available reducing buffer capacity may be important components of disease progression and biomarkers of clinical events.

### 4.3. Chronic Toxic Environment in MS Lesions

In smouldering lesions, despite the moderate inflammatory activity, there are clear signs of a toxic milieu that contributes to the progressive degeneration of neurons, axons and oligodendrocytes and, thus, to the clinical progression of MS. In addition to immune cells, the inflammatory process described above is thought to involve oxidative stress in MS lesions, the development of mitochondrial damage and consequent energy-deficient hypoxia, and iron accumulation. In addition to classical immune system mediators, this chronic toxic environment contains high concentrations of oxidants and iron ions, as well as glutamate, an excitatory neurotransmitter. These mechanisms appear early and play a major role in the development of disease progression, persistent neurological deficits and disability. In this chapter, we will discuss how these events, alone or in combination, lead to the destruction of neurons, axons and glial cells, ultimately leading to the process of neurodegeneration.

#### 4.3.1. Glutamate Excitotoxicity

Excitotoxicity is a pathological process in which over-activation of excitatory amino acid receptors leads to neuronal damage and death. The main mechanisms mediated by acute inflammation are excitotoxicity induced by the neurotransmitter glutamate. It causes cell death mainly by direct cytotoxicity or by increasing the intracellular calcium (Ca^2+^) concentration. In addition to molecular biological studies, pathophysiological studies, CSF analysis and in vivo studies (MR spectroscopy) have all shown that glutamate homeostasis in the brain is disturbed in MS. Particularly active lesions and even normal-appearing white matter show elevated glutamate concentrations, which may predict the extent of neuroaxonal damage [117,118]. In patients with progressive MS, lower levels of gamma-aminobutyric acid (GABA, the product of glutamate) in the sensory and motor cortex were associated with reduced motor performance, suggesting that this metabolite may also play a role in degenerative processes [119]. The main sources of extracellular glutamate are activated microglia/macrophage cells and leukocytes [120], but the exact source and distribution of glutamate are not clear. Inflammation is triggered by the release of large amounts of glutamate by activated microglia cells (which are activated in all subtypes of MS) [121], ultimately leading to neuronal excitotoxicity and death [120,122]. The N-methyl-D-aspartate (NMDA) receptor is central to glutamate excitotoxicity [123]. In the CNS, these receptors are found on the surface of neurons, astrocytes, oligodendrocytes and microglia [124]. QUIN (quinolinic acid) is known to be a selective agonist of NMDA receptors and is also an endogenous excitotoxin [125] and a neurotoxic metabolite of kynurenine [113]. QUIN increases synaptic glutamate release, inhibits reuptake and reduces the conversion of glutamate to glutamine. These effects increase synaptic glutamate concentrations and lead to excitotoxicity via overstimulation of NMDA receptors [126,127,128], resulting in elevated intracellular calcium levels. The consequence of elevated intracellular Ca^2+^ levels is an increase in the activation of several enzymes, which induces lipid peroxidation [129] through the formation of free radicals and mitochondrial dysfunction, leading to the production of ROS, oxidative stress and ultimately, apoptotic cell death [130,131]. This induced cell death has been observed under in vitro conditions at pathological concentrations of QUIN in human neurons, astrocytes, motor neurons and rat oligodendrocytes [132,133,134,135].

#### 4.3.2. Kynurenines

**Figure 4:**
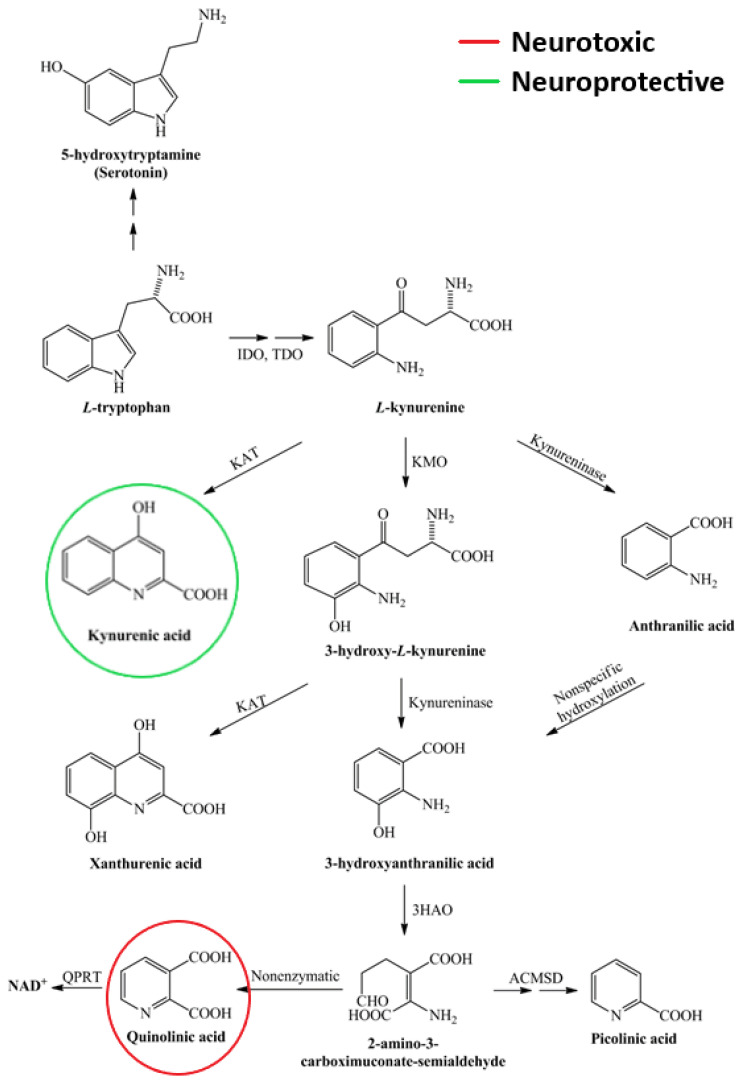
Scheme of the kynurenine pathway. As an essential amino acid, tryptophan is mainly catabolised via the kynurenine pathway, leading to the production of neurotoxic and neuroprotective metabolites. 3-HAO—3-hydroxyanthranilate oxidase; ACMSD—α-amino-β-carboxymuconate-semialdehyde-decarboxylase; IDO—indoleamine 2,3-dioxygenase; KAT—kynurenine aminotransferase; KMO—kynurenine 3-monooxygenase; NAD+—nicotinamide adenine dinucleotide; QPRT—quinolinate phosphoribosyltransferase; TDO—tryptophan 2,3-dioxygenase.

Kynurenine metabolism is the defining process of tryptophan metabolism (see Figure 4). Pro-inflammatory cytokines can influence the activation of indoleamine 2,3-dioxygenase (IDO-1) and thus significantly modify this process. The molecules produced during metabolism have neurotoxic (quinolinic acid, QUIN) and neuroprotective (kynurenic acid, KYNA) effects in addition to their immune inhibitory function (see reviews [113,136]). Studies by Lim et al. have demonstrated a significant shift in kynurenine metabolism in the blood of MS patients, particularly in the concentration of the latter two molecules. They suggest that changes in kynurenine metabolism may be an important biomarker in the transition from early mild to progressive forms of the disease [137]. Our recent studies have shown a correlation between changes in QUIN and neurofilament light chain (NfL) concentrations [138]. A potential criticism may be that biomarkers measured in the blood do not necessarily correspond to changes in the CNS. However, data from Lim et al. showed a very good correlation between changes in serum and CSF metabolite concentrations. The results to date suggest that during acute neuroinflammation, the production of neuroprotective kynurenine metabolites (KYNA) is predominant, most likely counteracting the effects of neurotoxic metabolites. As the disease progresses, the profile of the kynurenine pathway (KP) changes, and the chronic activation of the enzymes involved in the pathway increases the production of neurotoxic metabolites. It thus contributes to the development of multiple sclerosis [113]. In our recently published research, we found that MS patients with lower serum levels of 3-hydroxykynurenine (3HK) have higher levels of microglial activation. This finding is surprising because 3HK is known to promote neuronal apoptosis and increase oxidative stress under experimental conditions. We suggest that the correlation may be due to the variability in the levels of KP enzymes and metabolites in different body compartments [139]. Recently we found that the patented kynurenic acid analogue SZR104 induces cytomorphological changes associated with the anti-inflammatory phenotype in cultured microglia [140,141]. It is no coincidence that, as mentioned above, PET studies are underway to monitor the activity of IDO-1 and KMO in order to map kynurenine metabolism.

#### 4.3.3. Mitochondrial Dysfunction

It is becoming increasingly clear that the inflammatory process in MS leads to axonal/neuronal degeneration due to neuronal mitochondrial dysfunction, energy depletion and altered ion balance. Ion channels, through which Ca^2+^ and Na^+^ ions continuously flow into axons/neurons, are mainly involved in this process. The proteomic data showed a significant reduction, particularly in components associated with mitochondrial energy metabolism, in neurons affected by both acute and chronic lesions [142]. Studies in MS patients suggest that mitochondrial energy production in axons is reduced. In addition, energy demand is increased due to the increased expression of Na^+^ channels along the entire length of the demyelinated axolemma. The disturbance in ion balance leads to an increase in intracellular Ca^2+^ levels, and the resulting mitochondrial gene expression and functional changes trigger degenerative mechanisms [143,144]. In acute inflammatory lesions, the process of mitochondrial damage is initiated by soluble mediators produced by immune cells [145]. In the chronic stage of the disease, an accumulation of deletions in the mitochondrial genome has been demonstrated, which leads to mitochondrial dysfunction and may contribute to progressive neurodegeneration [146]. This mitochondrial damage not only occurs in neurons but also affects oligodendrocytes and their progenitors, impairing the ability of progenitors to differentiate into myelinating oligodendrocytes and thus may contribute to the failure of remyelination [147]. Using proton MR spectroscopy (H-MRS), reduced levels of NAA (N-acetylaspartic acid) and choline and increased levels of myo-inositol have been observed in intact brain tissue from MS patients, suggesting axonal damage, glial cell activity and increased cell membrane metabolism. The level of NAA is an indicator of neuronal number and viability. Although its biochemical function is not fully understood, it is likely to have a bioenergetic role in mitochondria and thus may well characterise the structural and energetic integrity of neurons. Acute lesions show reduced NAA concentrations and elevated choline and lactate levels. Elevated choline indicates membrane phospholipids released as a result of damage to the myelin sheath, and lactate indicates increased metabolism of cells involved in the inflammatory process. Increased levels of NAA in lesions are associated with clinical improvement, which in MS may support the theory that increased mitochondrial function is required for remyelination. In chronic lesions, reduced NAA levels indicate irreversible axonal damage. Furthermore, reduced NAA levels have also been found in patients with radiologically isolated syndrome (RIS), suggesting that axonal damage may be evident at a very early stage of the disease [148]. The persistence of a relatively low energy state eventually leads to chronic degeneration of axons in the brains of MS patients. Axonal degeneration only progresses in the presence of other toxic products produced during acute inflammation. One such toxic factor is oxidative stress.

#### 4.3.4. Essential Metal Homeostasis Disruption in MS

Improper handling or increased accumulation of essential metals such as manganese, iron and copper have been reported to exert neurotoxic effects in several neurological disorders [149]. A prominent example is chronic exposure to manganese, which can lead to a Parkinson-like syndrome called “manganism” [150]. Overexposure to this metal can cause several neurotoxic effects, including disruption of mitochondrial function, neurotransmitter metabolism, alteration of iron homeostasis and induction of oxidative stress [151]. In addition to manganese, other metal ions such as zinc, iron and copper have been implicated in the aggregation of Aβ-amyloid, α-synuclein or prion protein, thereby acting as triggers for neurodegeneration [152]. Iron and copper are of particular interest because they can catalyse the production of toxic ROS through Fenton chemistry [153]. In these reactions, reduced forms of iron and copper contribute to the generation of hydroxyl radicals, which cause oxidative damage to proteins, DNA and lipids.

Copper plays a central role in the cuprizone animal model of MS. In this model, the application of a copper chelator induces neurotoxic effects, specifically leading to the death of oligodendrocytes and subsequent demyelination, reflecting key features of MS pathology [154]. Recent evidence suggests that copper levels may play a role in modulating the switch between anti-inflammatory and pro-inflammatory phenotypes in microglia. This regulatory process is thought to occur via the modulation of NO levels and disruption of S-nitrosothiol signalling, both of which may have significant implications for the inflammatory aspects of MS [155].

**Figure 5:**
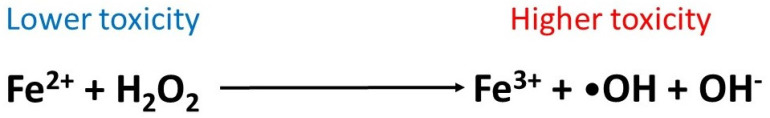
Fe^2+^ (free iron) reacts through the Fenton reaction with hydrogen peroxide (H_2_O_2_), leading to the generation of highly reactive and damaging hydroxyl radicals (OH•). Highly reactive free hydroxyl radicals interact with molecules leading to the production of other free radicals, which then lead to oxidative stress-induced mitochondrial dysfunction, lipid peroxidation, DNA damage and, ultimately, cell dysfunction, and death.

The process of oxidative injury is further enhanced during the inflammatory process by the accumulation of iron in the ageing human brain, particularly in oligodendrocytes, macrophages and microglia cells [156]. The toxic effect of iron ions is largely inhibited when they are bound to the intracellular protein ferritin. However, the destruction of oligodendrocytes and microglia results in the release of free Fe^2+^ ions into the extracellular space [95]. Due to the Fenton reaction (during which Fe^2+^ is transformed into Fe^3+^ ions) (see Figure 5), iron ions can actively promote the formation of highly active hydroxyl radicals, leading to further damage [157]. As mentioned above, smouldering lesions are predominantly composed of a loose network of iron-loaded activated microglia cells and macrophages. The fact that the increase in the volume of smouldering lesions is not observed in similar lesions without an iron ring suggests that iron deposition and release contribute to the maintenance of the chronic inflammatory response in the brain of MS patients. Metals can also bind to the sulfhydryl group (-SH) in enzymes, coenzymes, and proteins, inducing dysfunctions in the immune system [158]. Therefore, understanding and exploring the role of metal dysregulation may provide promising avenues for therapeutic intervention in MS.

## 5. PIRA and Future Perspectives of MS Research

MS is a complex disease at the interface of immunology and neurobiology. Simultaneous focal and diffuse tissue damage can be demonstrated as a consequence of inflammation and neurodegeneration. Initially, the effects of focal inflammation may be functionally compensated for in the patient, depending on the extent of pre-existing CNS damage, the reserve capacity of the brain, and the ability of the brain to restore function or compensate for the damage caused. This explains why most of the focal inflammatory lesions detected by MRI in the early stages of the disease are undetected by the patient, who recovers from relapses corresponding to clinical manifestations of inflammatory activity without residual symptoms. However, the latently progressive neurodegenerative process causes irreversible damage to the CNS and leads to the development of progressive neurological dysfunction. The latest terminology refers to this process as PIRA. The neuroinflammatory-neurodegenerative process, which causes progression independent of relapse activity, is reflected in the development of smouldering lesions and the accelerated brain and spinal cord atrophy seen on MRI images, which can be detected in the early phase of the disease and correlates closely with long-term prognosis [14]. Several different mechanisms may contribute to the development of PIRA. Some of these are undoubtedly the result of focal inflammatory lesions, while others are thought to be independent.

The early progression of the disease, independent of relapses, draws attention to the partial discrepancy between anti-inflammatory therapy and disease progression. Immunomodulatory or immunosuppressive therapies remain at the forefront of the therapeutic armamentarium. In the future, neuroprotective therapies (see Table 1) will be increasingly in demand. In addition, as the disease progresses, the inflammatory response appears to retreat behind the BBB and is no longer accessible to immunosuppressive agents. In the future, the treatment of MS will probably require a combination of therapies that simultaneously target peripheral immune cell function and inflammation within the CNS at an early stage and that also have a neuroprotective or remyelination-promoting neuroregenerative mechanism of action.

The current classification, as mentioned above, divides progressive MS into a secondary progressive (SPMS) and primary progressive (PPMS) course. The relevance of this classification is not only semantic but has important clinical implications as it affects how clinical trials are conducted and whether their results can be applied interchangeably between these forms of MS. The main difference between the two is whether there is a relapsing-remitting phase (SPMS) or not (PPMS). Long-term follow-up of patients with RIS has shown that both relapsing-remitting MS and PPMS can develop [175]. Furthermore, pathological analyses suggest that the differences between SPMS and PPMS are quantitative rather than qualitative [9,11]. As mentioned above, it is a fact that inflammation and neurodegeneration persist in all MS patients regardless of clinical phenotype and disease stage. This evidence suggests that SPMS and PPMS may, in fact, be part of the same disease spectrum. It may seem a bold claim, but the above data suggest that there is no real biological basis for a sharp distinction between different MS phenotypes. We argue that PPMS may simply be a form of SPMS in which the relapsing-remitting phase is clinically silent. This leads to the conclusion that the categorisation into SPMS and PPMS based on their pathogenesis seems arbitrary and may not be of much relevance.

It has already been mentioned that there is evidence of many similar pathological changes in relapsing-remitting and progressive MS, which are mainly quantitative rather than qualitative in nature. The transition from a relapsing-remitting course to a progressive course seems to mark the point at which the brain’s reserves are exhausted and can no longer compensate for axonal loss. It is also possible that PPMS is actually preceded by an “asymptomatic (or unrecognised)” relapsing-remitting (RR) phase of the disease, depending on the predominance of an adaptive immune response. These data reinforce the unified view of MS and raise the possibility that the disease is, in fact, a primary progressive pathology with superimposed relapses. Therefore, the medical community caring for MS patients is faced with a major change in perspective that may influence the future assessment of the clinical course of MS and therapeutic decisions.

Another important question is the identity of the MS-specific inflammatory cell-derived soluble factor that drives demyelination and neurodegeneration, directly or indirectly inducing tissue damage through microglial activation. We do not know yet whether combined treatment will be feasible in the future. BTK inhibitors appear to be a promising therapy for MS. However, their effect on SELs is still uncertain because of the short follow-up period. In the future, when a more accurate picture of the role of infectious agents in the pathomechanism of MS becomes available, a programme of MS therapy with a radically new approach may be developed. There are still several unanswered questions that may be answered by future research.

## 6. Conclusions

In this review, we have detailed how, in addition to classical immune system mediators, various pathobiochemical markers and mechanisms, alone or in combination, lead to the destruction of neurons, axons and glial cells, ultimately leading to a process of neuroinflammation and neurodegeneration in MS. As described above, from a biological point of view, this is a single process. These new immunological and pathological findings are changing our understanding of MS, which is no longer seen as a two-stage disease but rather as a continuum in which both inflammation and neurodegeneration are simultaneously present at any point in the disease course. In many ways, this new approach to MS poses a serious challenge to the current clinical classification of the disease and draws attention to new therapeutic targets.

## Figures and Tables

**Table 1:** Potential neuroprotective therapies in MS.

Product Name	Pharmacological Actions and Mechanisms	References
Simvastatin	Pleiotropic effects, including modulation of excitotoxicity	[159]
N-acetyl cysteine	Glutathione (GSH) precursor with antioxidant properties	[160]
Ketamine	Glutamate antagonists	[161]
Clemastine fumarate	Antihistamine	[162]
Minocycline	Second-generation tetracycline antibiotic with immunomodulating properties	[163,164]
Ibudilast	Anti-apoptotic agent (non-selective phosphodiesterase inhibitor)	[165]
Bruton’s tyrosine kinase inhibitors (BTKi)	Agent acting on microglia	[166]
Testosterone	Immunomodulatory effects	[167]
Basic fibroblast growth factor (bFGF)	Promotes proliferation and migration of OPCs	[168]
Erythropoietin (EPO)	Blocking of ROS production and related apoptosis, neuroprotective effects, and stimulation of neurogenesis	[169,170]
Coenzyme Q10	Antioxidant agent	[171]
Idebenone	Synthetic analogue of coenzyme Q10	[172]
Mitoquinone (MitoQ)	Mitochondria-targeted antioxidant	[173]
Pioglitazone	Agonists of the peroxisome proliferator-activated receptors (PPARγ)	[174]
SZR104	Anti-inflammatory phenotype in cultured microglia	[140,141]

SZR104—N-(2-(dimethylamino)ethyl)-3-(morpholinomethyl)-4-hydroxyquinoline-2-carboxamide.

## References

[1] Lassmann H., Bruck W., Lucchinetti C.F. (2007). The immunopathology of multiple sclerosis: An overview. Brain Pathol..

[2] Biström M., Jons D., Engdahl E., Gustafsson R., Huang J., Brenner N., Butt J., Alonso-Magdalena L., Gunnarsson M., Vrethem M. (2020). Epstein–Barr virus infection after adolescence and human herpesvirus 6A as risk factors for multiple sclerosis. Eur. J. Neurol..

[3] Lublin F.D. (2014). New Multiple Sclerosis Phenotypic Classification. Eur. Neurol..

[4] Cree B.A., Hollenbach J.A., Bove R., Kirkish G., Sacco S., Caverzasi E., Bischof A., Gundel T., Zhu A.H., Papinutto N. (2019). Silent progression in disease activity-free relapsing multiple sclerosis. Ann. Neurol..

[5] Jonkman L.E., Soriano A.L., Amor S., Barkhof F., van der Valk P., Vrenken H., Geurts J.J.G. (2015). Can MS lesion stages be distinguished with MRI? A postmortem MRI and histopathology study. J. Neurol..

[6] Kipp M., van der Valk P., Amor S. (2012). Pathology of multiple sclerosis. CNS Neurol. Disord. Drug Targets.

[7] Haase S., Linker R.A. (2021). Inflammation in multiple sclerosis. Ther. Adv. Neurol. Disord..

[8] Machado-Santos J., Saji E., Tröscher A.R., Paunovic M., Liblau R., Gabriely G., Bien C.G., Bauer J., Lassmann H. (2018). The compartmentalized inflammatory response in the multiple sclerosis brain is composed of tissue-resident CD8+ T lymphocytes and B cells. Brain.

[9] Frischer J.M., Bramow S., Dal-Bianco A., Lucchinetti C.F., Rauschka H., Schmidbauer M., Laursen H., Sorensen P.S., Lassmann H. (2009). The relation between inflammation and neurodegeneration in multiple sclerosis brains. Brain J. Neurol..

[10] Kutzelnigg A., Lassmann H. (2014). Pathology of multiple sclerosis and related inflammatory demyelinating diseases. Handb. Clin. Neurol..

[11] Frischer J.M., Weigand S.D., Guo Y., Kale N., Parisi J.E., Pirko I., Mandrekar J., Bramow S., Metz I., Brück W. (2015). Clinical and pathological insights into the dynamic nature of the white matter multiple sclerosis plaque. Ann. Neurol..

[12] Absinta M., Sati P., Masuzzo F., Nair G., Sethi V., Kolb H., Ohayon J., Wu T., Cortese I.C.M., Reich D.S. (2019). Association of Chronic Active Multiple Sclerosis Lesions with Disability In Vivo. JAMA Neurol..

[13] Kuhlmann T., Ludwin S., Prat A., Antel J., Brück W., Lassmann H. (2016). An updated histological classification system for multiple sclerosis lesions. Acta Neuropathol..

[14] Giovannoni G., Popescu V., Wuerfel J., Hellwig K., Iacobaeus E., Jensen M.B., García-Domínguez J.M., Sousa L., De Rossi N., Hupperts R. (2022). Smouldering multiple sclerosis: The ‘real MS’. Ther. Adv. Neurol. Disord..

[15] Absinta M., Sati P., Fechner A., Schindler M., Nair G., Reich D. (2018). Identification of Chronic Active Multiple Sclerosis Lesions on 3T MRI. Am. J. Neuroradiol..

[16] Absinta M., Sati P., Schindler M., Leibovitch E.C., Ohayon J., Wu T., Meani A., Filippi M., Jacobson S., Cortese I.C. (2016). Persistent 7-tesla phase rim predicts poor outcome in new multiple sclerosis patient lesions. J. Clin. Investig..

[17] Hemond C.C., Reich D.S., Dundamadappa S.K. (2022). Paramagnetic Rim Lesions in Multiple Sclerosis: Comparison of Visualization at 1.5-T and 3-T MRI. Am. J. Roentgenol..

[18] Calvi A., Haider L., Prados F., Tur C., Chard D., Barkhof F. (2020). In vivo imaging of chronic active lesions in multiple sclerosis. Mult. Scler. J..

[19] Elliott C., Wolinsky J.S., Hauser S.L., Kappos L., Barkhof F., Bernasconi C., Wei W., Belachew S., Arnold D.L. (2018). Slowly expanding/evolving lesions as a magnetic resonance imaging marker of chronic active multiple sclerosis lesions. Mult. Scler. J..

[20] Bagnato F., Hametner S., Yao B., Van Gelderen P., Merkle H., Cantor F.K., Lassmann H., Duyn J.H. (2011). Tracking iron in multiple sclerosis: A combined imaging and histopathological study at 7 Tesla. Brain.

[21] Dal-Bianco A., Grabner G., Kronnerwetter C., Weber M., Höftberger R., Berger T., Auff E., Leutmezer F., Trattnig S., Lassmann H. (2016). Slow expansion of multiple sclerosis iron rim lesions: Pathology and 7 T magnetic resonance imaging. Acta Neuropathol..

[22] Arnold D.L., Belachew S., Gafson A.R., Gaetano L., Bernasconi C., Elliott C. (2021). Slowly expanding lesions are a marker of progressive MS—No. Mult. Scler. J..

[23] Altokhis A.I., Hibbert A.M., Allen C.M., Mougin O., Alotaibi A., Lim S.-Y., Constantinescu C.S., Abdel-Fahim R., Evangelou N. (2022). Longitudinal clinical study of patients with iron rim lesions in multiple sclerosis. Mult. Scler. J..

[24] Preziosa P., Pagani E., Meani A., Moiola L., Rodegher M., Filippi M., Rocca M.A. (2022). Slowly Expanding Lesions Predict 9-Year Multiple Sclerosis Disease Progression. Neurol.-Neuroimmunol. Neuroinflamm..

[25] Koch M.W., Metz L.M., Agrawal S.M., Yong V.W. (2013). Environmental factors and their regulation of immunity in multiple sclerosis. J. Neurol. Sci..

[26] Lassmann H., Bradl M. (2016). Multiple sclerosis: Experimental models and reality. Acta Neuropathol..

[27] Lucchinetti C.F., Popescu B.F., Bunyan R.F., Moll N.M., Roemer S.F., Lassmann H., Brück W., Parisi J.E., Scheithauer B.W., Giannini C. (2011). Inflammatory cortical demyelination in early multiple sclerosis. N. Engl. J. Med..

[28] Smolders J., Heutinck K.M., Fransen N.L., Remmerswaal E.B.M., Hombrink P., Berge I.J.M.T., van Lier R.A.W., Huitinga I., Hamann J. (2018). Tissue-resident memory T cells populate the human brain. Nat. Commun..

[29] Van Nierop G.P., van Luijn M.M., Michels S.S., Melief M.-J., Janssen M., Langerak A.W., Ouwendijk W.J.D., Hintzen R.Q., Verjans G.M.G.M. (2017). Phenotypic and functional characterization of T cells in white matter lesions of multiple sclerosis patients. Acta Neuropathol..

[30] Babbe H., Roers A., Waisman A., Lassmann H., Goebels N., Hohlfeld R., Friese M., Schröder R., Deckert M., Schmidt S. (2000). Clonal Expansions of Cd8+ T Cells Dominate the T Cell Infiltrate in Active Multiple Sclerosis Lesions as Shown by Micromanipulation and Single Cell Polymerase Chain Reaction. J. Exp. Med..

[31] Denic A., Wootla B., Rodriguez M. (2013). CD8(+) T cells in multiple sclerosis. Expert Opin. Ther. Targets.

[32] Vukmanovic-Stejic M., Thomas M.J., Noble A., Kemeny D.M. (2001). Specificity, restriction and effector mechanisms of immunoregulatory CD8 T cells. Immunology.

[33] Jadidi-Niaragh F., Mirshafiey A. (2011). Th17 Cell, the New Player of Neuroinflammatory Process in Multiple Sclerosis. Scand. J. Immunol..

[34] Ramgolam V.S., Sha Y., Jin J., Zhang X., Markovic-Plese S. (2009). IFN-beta inhibits human Th17 cell differentiation. J. Immunol..

[35] Zhang X., Markovic-Plese S. (2010). Interferon beta inhibits the Th17 cell-mediated autoimmune response in patients with relapsing–remitting multiple sclerosis. Clin. Neurol. Neurosurg..

[36] Zozulya A.L., Wiendl H. (2008). The role of regulatory T cells in multiple sclerosis. Nat. Clin. Pr. Neurol..

[37] Chen M., Chen G., Deng S., Liu X., Hutton G.J., Hong J. (2012). IFN-β induces the proliferation of CD4+CD25+Foxp3+ regulatory T cells through upregulation of GITRL on dendritic cells in the treatment of multiple sclerosis. J. Neuroimmunol..

[38] Haas J., Korporal M., Balint B., Fritzsching B., Schwarz A., Wildemann B. (2009). Glatiramer acetate improves regulatory T-cell function by expansion of naive CD4+CD25+FOXP3+CD31+ T-cells in patients with multiple sclerosis. J. Neuroimmunol..

[39] Haas J., Würthwein C., Korporal-Kuhnke M., Viehoever A., Jarius S., Ruck T., Pfeuffer S., Meuth S.G., Wildemann B. (2019). Alemtuzumab in Multiple Sclerosis: Short- and Long-Term Effects of Immunodepletion on the Peripheral Treg Compartment. Front. Immunol..

[40] Trinschek B., Luessi F., Gross C.C., Wiendl H., Jonuleit H. (2015). Interferon-Beta Therapy of Multiple Sclerosis Patients Improves the Responsiveness of T Cells for Immune Suppression by Regulatory T Cells. Int. J. Mol. Sci..

[41] Krienke C., Kolb L., Diken E., Streuber M., Kirchhoff S., Bukur T., Akilli-Öztürk Ö., Kranz L.M., Berger H., Petschenka J. (2021). A noninflammatory mRNA vaccine for treatment of experimental autoimmune encephalomyelitis. Science.

[42] Miyazaki Y., Niino M. (2021). B-cell depletion therapy for multiple sclerosis. Immunol. Med..

[43] Baker D., Marta M., Pryce G., Giovannoni G., Schmierer K. (2017). Memory B Cells are Major Targets for Effective Immunotherapy in Relapsing Multiple Sclerosis. Ebiomedicine.

[44] Dobson R., Ramagopalan S., Davis A., Giovannoni G. (2013). Cerebrospinal fluid oligoclonal bands in multiple sclerosis and clinically isolated syndromes: A meta-analysis of prevalence, prognosis and effect of latitude. J. Neurol. Neurosurg. Psychiatry.

[45] Mulero P., Midaglia L., Montalban X. (2018). Ocrelizumab: A new milestone in multiple sclerosis therapy. Ther. Adv. Neurol. Disord..

[46] Bar-Or A., Fawaz L., Fan B., Darlington P.J., Rieger A., Ghorayeb C., Calabresi P.A., Waubant E., Hauser S.L., Zhang J. (2010). Abnormal B-cell cytokine responses a trigger of T-cell-mediated disease in MS?. Ann. Neurol..

[47] Howell O.W., Reeves C.A., Nicholas R., Carassiti D., Radotra B., Gentleman S.M., Serafini B., Aloisi F., Roncaroli F., Magliozzi R. (2011). Meningeal inflammation is widespread and linked to cortical pathology in multiple sclerosis. Brain.

[48] Magliozzi R., Howell O.W., Reeves C., Roncaroli F., Nicholas R., Serafini B., Aloisi F., Reynolds R. (2010). A Gradient of neuronal loss and meningeal inflammation in multiple sclerosis. Ann. Neurol..

[49] Serafini B., Rosicarelli B., Magliozzi R., Stigliano E., Aloisi F. (2004). Detection of Ectopic B-cell Follicles with Germinal Centers in the Meninges of Patients with Secondary Progressive Multiple Sclerosis. Brain Pathol..

[50] Lisak R.P., Benjamins J.A., Nedelkoska L., Barger J.L., Ragheb S., Fan B., Ouamara N., Johnson T.A., Rajasekharan S., Bar-Or A. (2012). Secretory products of multiple sclerosis B cells are cytotoxic to oligodendroglia in vitro. J. Neuroimmunol..

[51] Owens G.P., Bennett J.L., Lassmann H., O’Connor K.C., Ritchie A.M., Shearer A., Lam C., Yu X., Birlea M., DuPree C. (2009). Antibodies produced by clonally expanded plasma cells in multiple sclerosis cerebrospinal fluid. Ann. Neurol..

[52] Kumar G., Axtell R.C. (2023). Dual Role of B Cells in Multiple Sclerosis. Int. J. Mol. Sci..

[53] Ginhoux F., Lim S., Hoeffel G., Low D., Huber T. (2013). Origin and differentiation of microglia. Front. Cell Neurosci..

[54] Kreutzberg G.W. (1996). Microglia: A sensor for pathological events in the CNS. Trends Neurosci..

[55] Perry V.H., Teeling J. (2013). Microglia and macrophages of the central nervous system: The contribution of microglia priming and systemic inflammation to chronic neurodegeneration. Semin. Immunopathol..

[56] Kettenmann H., Hanisch U.-K., Noda M., Verkhratsky A. (2011). Physiology of Microglia. Physiol. Rev..

[57] Chhor V., Le Charpentier T., Lebon S., Oré M.-V., Celador I.L., Josserand J., Degos V., Jacotot E., Hagberg H., Sävman K. (2013). Characterization of phenotype markers and neuronotoxic potential of polarised primary microglia in vitro. Brain Behav. Immun..

[58] Colton C.A., Gilbert D.L. (1987). Production of superoxide anions by a CNS macrophage, the microglia. FEBS Lett..

[59] Ding A.H., Nathan C.F., Stuehr D.J. (1988). Release of reactive nitrogen intermediates and reactive oxygen intermediates from mouse peritoneal macrophages. Comparison of activating cytokines and evidence for independent production. J. Immunol..

[60] Martinez F.O., Sica A., Mantovani A., Locati M. (2008). Macrophage activation and polarization. Front. Biosci. J. Virtual Libr..

[61] Böttcher C., van der Poel M., Fernández-Zapata C., Schlickeiser S., Leman J.K.H., Hsiao C.-C., Mizee M.R., Adelia, Vincenten M.C.J., Kunkel D. (2020). Single-cell mass cytometry reveals complex myeloid cell composition in active lesions of progressive multiple sclerosis. Acta Neuropathol. Commun..

[62] O’loughlin E., Madore C., Lassmann H., Butovsky O. (2018). Microglial Phenotypes and Functions in Multiple Sclerosis. Cold Spring Harb. Perspect. Med..

[63] Liddelow S.A., Guttenplan K.A., Clarke L.E., Bennett F.C., Bohlen C.J., Schirmer L., Bennett M.L., Münch A.E., Chung W.S., Peterson T.C. (2017). Neurotoxic reactive astrocytes are induced by activated microglia. Nature.

[64] Rothhammer V., Borucki D.M., Tjon E.C., Takenaka M.C., Chao C.C., Ardura-Fabregat A., De Lima K.A., Gutiérrez-Vázquez C., Hewson P., Staszewski O. (2018). Microglial control of astrocytes in response to microbial metabolites. Nature.

[65] Lawrence J.M., Schardien K., Wigdahl B., Nonnemacher M.R. (2023). Roles of neuropathology-associated reactive astrocytes: A systematic review. Acta Neuropathol. Commun..

[66] Absinta M., Maric D., Gharagozloo M., Garton T., Smith M.D., Jin J., Fitzgerald K.C., Song A., Liu P., Lin J.P. (2021). A lymphocyte-microglia-astrocyte axis in chronic active multiple sclerosis. Nature.

[67] Fischer M.-T., Sharma R., Lim J.L., Haider L., Frischer J.M., Drexhage J., Mahad D., Bradl M., Van Horssen J., Lassmann H. (2012). NADPH oxidase expression in active multiple sclerosis lesions in relation to oxidative tissue damage and mitochondrial injury. Brain.

[68] Lisak R.P., Benjamins J.A., Bealmear B., Nedelkoska L., Studzinski D., Retland E., Yao B., Land S. (2009). Differential effects of Th1, monocyte/macrophage and Th2 cytokine mixtures on early gene expression for molecules associated with metabolism, signaling and regulation in central nervous system mixed glial cell cultures. J. Neuroinflamm..

[69] Hametner S., Wimmer I., Haider L., Pfeifenbring S., Brück W., Lassmann H. (2013). Iron and neurodegeneration in the multiple sclerosis brain. Ann. Neurol..

[70] Gao Z., Tsirka S.E. (2011). Animal Models of MS Reveal Multiple Roles of Microglia in Disease Pathogenesis. Neurol. Res. Int..

[71] McMahon E.J., Bailey S.L., Castenada C.V., Waldner H., Miller S.D. (2005). Epitope spreading initiates in the CNS in two mouse models of multiple sclerosis. Nat. Med..

[72] Krumbholz M., Theil D., Derfuss T., Rosenwald A., Schrader F., Monoranu C.-M., Kalled S.L., Hess D.M., Serafini B., Aloisi F. (2005). BAFF is produced by astrocytes and up-regulated in multiple sclerosis lesions and primary central nervous system lymphoma. J. Exp. Med..

[73] Breij E.C.W., Brink B.P., Veerhuis R., Bsc C.v.D.B., Vloet R., Yan R., Dijkstra C.D., van der Valk P., Bö L. (2008). Homogeneity of active demyelinating lesions in established multiple sclerosis. Ann. Neurol..

[74] Kutzelnigg A., Lucchinetti C.F., Stadelmann C., Brück W., Rauschka H., Bergmann M., Schmidbauer M., Parisi J.E., Lassmann H. (2005). Cortical demyelination and diffuse white matter injury in multiple sclerosis. Brain J. Neurol..

[75] Airas L., Nylund M., Rissanen E. (2018). Evaluation of Microglial Activation in Multiple Sclerosis Patients Using Positron Emission Tomography. Front. Neurol..

[76] Karamita M., Barnum C., Möbius W., Tansey M.G., Szymkowski D.E., Lassmann H., Probert L. (2017). Therapeutic inhibition of soluble brain TNF promotes remyelination by increasing myelin phagocytosis by microglia. J. Clin. Investig..

[77] Lampron A., Larochelle A., Laflamme N., Préfontaine P., Plante M.-M., Sánchez M.G., Yong V.W., Stys P.K., Tremblay M., Rivest S. (2015). Inefficient clearance of myelin debris by microglia impairs remyelinating processes. J. Exp. Med..

[78] Poliani P.L., Wang Y., Fontana E., Robinette M.L., Yamanishi Y., Gilfillan S., Colonna M. (2015). TREM2 sustains microglial expansion during aging and response to demyelination. J. Clin. Investig..

[79] Yamasaki R., Lu H., Butovsky O., Ohno N., Rietsch A.M., Cialic R., Wu P.M., Doykan C.E., Lin J., Cotleur A.C. (2014). Differential roles of microglia and monocytes in the inflamed central nervous system. J. Exp. Med..

[80] Boyd A., Zhang H., Williams A. (2013). Insufficient OPC migration into demyelinated lesions is a cause of poor remyelination in MS and mouse models. Acta Neuropathol..

[81] Franklin R.J., Goldman S.A. (2015). Glia Disease and Repair-Remyelination. Cold Spring Harb. Perspect. Biol..

[82] Miron V.E., Boyd A., Zhao J.-W., Yuen T.J., Ruckh J.M., Shadrach J.L., van Wijngaarden P., Wagers A.J., Williams A., Franklin R.J.M. (2013). M2 microglia and macrophages drive oligodendrocyte differentiation during CNS remyelination. Nat. Neurosci..

[83] Mado H., Adamczyk-Sowa M., Sowa P. (2023). Role of Microglial Cells in the Pathophysiology of MS: Synergistic or Antagonistic?. Int. J. Mol. Sci..

[84] Veremeyko T., Siddiqui S., Sotnikov I., Yung A., Ponomarev E.D. (2013). IL-4/IL-13-dependent and independent expression of miR-124 and its contribution to M2 phenotype of monocytic cells in normal conditions and during allergic inflammation. PLoS ONE.

[85] Zhang L., Zhang J., You Z. (2018). Switching of the Microglial Activation Phenotype Is a Possible Treatment for Depression Disorder. Front. Cell. Neurosci..

[86] Lloyd A.F., Davies C.L., Holloway R.K., Labrak Y., Ireland G., Carradori D., Dillenburg A., Borger E., Soong D., Richardson J.C. (2019). Central nervous system regeneration is driven by microglia necroptosis and repopulation. Nat. Neurosci..

[87] Gao H.-M., Hong J.-S. (2008). Why neurodegenerative diseases are progressive: Uncontrolled inflammation drives disease progression. Trends Immunol..

[88] Howell O.W., Rundle J.L., Garg A., Komada M., Brophy P.J., Reynolds R. (2010). Activated Microglia Mediate Axoglial Disruption That Contributes to Axonal Injury in Multiple Sclerosis. J. Neuropathol. Exp. Neurol..

[89] Milo R., Korczyn A.D., Manouchehri N., Stüve O. (2019). The temporal and causal relationship between inflammation and neurodegeneration in multiple sclerosis. Mult. Scler. J..

[90] Calahorra L., Camacho-Toledano C., Serrano-Regal M.P., Ortega M.C., Clemente D. (2022). Regulatory Cells in Multiple Sclerosis: From Blood to Brain. Biomedicines.

[91] Kutzelnigg A., Lassmann H. (2006). Cortical demyelination in multiple sclerosis: A substrate for cognitive deficits?. J. Neurol. Sci..

[92] Lucchinetti C., Brück W., Parisi J., Scheithauer B., Rodriguez M., Lassmann H. (2000). Heterogeneity of multiple sclerosis lesions: Implications for the pathogenesis of demyelination. Ann. Neurol..

[93] Aloisi F., Pujol-Borrell R. (2006). Lymphoid neogenesis in chronic inflammatory diseases. Nat. Rev. Immunol..

[94] Esiri M., Gay D. (1990). Immunological and neuropathological significance of the Virchow-Robin space. J. Neurol. Sci..

[95] Lassmann H., van Horssen J., Mahad D. (2012). Progressive multiple sclerosis: Pathology and pathogenesis. Nat. Rev. Neurol..

[96] Magliozzi R., Howell O., Vora A., Serafini B., Nicholas R., Puopolo M., Reynolds R., Aloisi F. (2007). Meningeal B-cell follicles in secondary progressive multiple sclerosis associate with early onset of disease and severe cortical pathology. Brain J. Neurol..

[97] Makshakov G., Magonov E., Totolyan N., Nazarov V., Lapin S., Mazing A., Verbitskaya E., Trofimova T., Krasnov V., Shumilina M. (2017). Leptomeningeal Contrast Enhancement Is Associated with Disability Progression and Grey Matter Atrophy in Multiple Sclerosis. Neurol. Res. Int..

[98] Zivadinov R., Ramasamy D.P., Vaneckova M., Gandhi S., Chandra A., Hagemeier J., Bergsland N., Polak P., Benedict R.H., Hojnacki D. (2016). Leptomeningeal contrast enhancement is associated with progression of cortical atrophy in MS: A retrospective, pilot, observational longitudinal study. Mult. Scler. J..

[99] Fransen N.L., de Jong B.A., Heß K., Kuhlmann T., Vincenten M.C., Hamann J., Huitinga I., Smolders J. (2021). Absence of B Cells in Brainstem and White Matter Lesions Associates with Less Severe Disease and Absence of Oligoclonal Bands in MS. Neurol.-Neuroimmunol. Neuroinflamm..

[100] Guerrero B.L., Sicotte N.L. (2020). Microglia in Multiple Sclerosis: Friend or Foe?. Front. Immunol..

[101] Lassmann H. (2014). Multiple sclerosis: Lessons from molecular neuropathology. Exp. Neurol..

[102] De Groot C.J.A., Bergers E., Kamphorst W., Ravid R., Polman C.H., Barkhof F., van der Valk P. (2001). Post-mortem MRI-guided sampling of multiple sclerosis brain lesions: Increased yield of active demyelinating and (p)reactive lesions. Brain.

[103] Singh S., Metz I., Amor S., van der Valk P., Stadelmann C., Brück W. (2013). Microglial nodules in early multiple sclerosis white matter are associated with degenerating axons. Acta Neuropathol..

[104] Bedard K., Krause K.-H. (2007). The NOX Family of ROS-Generating NADPH Oxidases: Physiology and Pathophysiology. Physiol. Rev..

[105] van Horssen J., Witte M.E., Schreibelt G., de Vries H.E. (2011). Radical changes in multiple sclerosis pathogenesis. Biochim. Biophys. Acta (BBA)-Mol. Basis Dis..

[106] Smith K.J., Lassmann H. (2002). The role of nitric oxide in multiple sclerosis. Lancet Neurol..

[107] Colton C.A., Gilbert D.L. (1993). Microglia, an in vivo source of reactive oxygen species in the brain. Adv. Neurol..

[108] Smith K.J. (2011). Newly lesioned tissue in multiple sclerosis—A role for oxidative damage?. Brain J. Neurol..

[109] Braidy N., Grant R. (2017). Kynurenine pathway metabolism and neuroinflammatory disease. Neural Regen. Res..

[110] Murphy M.P. (1999). Nitric oxide and cell death. Biochim. Biophys. Acta.

[111] Martinelli R., Gegg M., Longbottom R., Adamson P., Turowski P., Greenwood J. (2009). ICAM-1–mediated Endothelial Nitric Oxide Synthase Activation via Calcium and AMP-activated Protein Kinase Is Required for Transendothelial Lymphocyte Migration. Mol. Biol. Cell.

[112] Karg E., Klivényi P., Németh I., Bencsik K., Pintér S., Vécsei L. (1999). Nonenzymatic antioxidants of blood in multiple sclerosis. J. Neurol..

[113] Pukoli D., Polyák H., Rajda C., Vécsei L. (2021). Kynurenines and Neurofilament Light Chain in Multiple Sclerosis. Front. Neurosci..

[114] Smerjac S.M., Bizzozero O.A. (2007). Cytoskeletal protein carbonylation and degradation in experimental autoimmune encephalomyelitis. J. Neurochem..

[115] Seven A., Aslan M., Incir S., Altıntaș A. (2013). Original article Evaluation of oxidative and nitrosative stress in relapsing remitting multiple sclerosis: Effect of corticosteroid therapy. Folia Neuropathol..

[116] Haider L., Zrzavy T., Hametner S., Höftberger R., Bagnato F., Grabner G., Trattnig S., Pfeifenbring S., Brück W., Lassmann H. (2016). The topograpy of demyelination and neurodegeneration in the multiple sclerosis brain. Brain.

[117] Azevedo C.J., Kornak J., Chu P., Sampat M., Okuda D.T., Cree B.A., Nelson S.J., Hauser S.L., Pelletier D. (2014). In vivo evidence of glutamate toxicity in multiple sclerosis. Ann. Neurol..

[118] Rajda C., Pukoli D., Bende Z., Majláth Z., Vécsei L. (2017). Excitotoxins, Mitochondrial and Redox Disturbances in Multiple Sclerosis. Int. J. Mol. Sci..

[119] Cawley N., Solanky B.S., Muhlert N., Tur C., Edden R.A.E., Wheeler-Kingshott C.A.M., Miller D.H., Thompson A.J., Ciccarelli O. (2015). Reduced gamma-aminobutyric acid concentration is associated with physical disability in progressive multiple sclerosis. Brain.

[120] Stojanovic I.R., Kostic M., Ljubisavljevic S. (2014). The role of glutamate and its receptors in multiple sclerosis. J. Neural Transm..

[121] Sriram S., Rodriguez M. (1997). Indictment of the microglia as the villain in multiple sclerosis. Neurology.

[122] Piani D., Frei K., Do K.Q., Cuénod M., Fontana A. (1991). Murine brain macrophages induce NMDA receptor mediated neurotoxicity in vitro by secreting glutamate. Neurosci. Lett..

[123] Fuchs S.A., Peeters-Scholte C.M.P.C.D., de Barse M.M.J., Roeleveld M.W., Klomp L.W.J., Berger R., de Koning T.J. (2011). Increased concentrations of both NMDA receptor co-agonists d-serine and glycine in global ischemia: A potential novel treatment target for perinatal asphyxia. Amino Acids.

[124] Kaindl A.M., Degos V., Peineau S., Gouadon E., Chhor V., Loron G., Le Charpentier T., Josserand J., Ali C., Vivien D. (2012). Activation of microglial N-methyl-D-aspartate receptors triggers inflammation and neuronal cell death in the developing and mature brain. Ann. Neurol..

[125] Stone T., Perkins M. (1981). Quinolinic acid: A potent endogenous excitant at amino acid receptors in CNS. Eur. J. Pharmacol..

[126] Tavares R.G., Tasca C.I., Santos C.E., Alves L.B., Porciúncula L.O., Emanuelli T., Souza D.O. (2002). Quinolinic acid stimulates synaptosomal glutamate release and inhibits glutamate uptake into astrocytes. Neurochem. Int..

[127] Tavares R.G., Tasca C.I., Santos C.E.S., Wajner M., Souza D., Dutra-Filho C. (2000). Quinolinic acid inhibits glutamate uptake into synaptic vesicles from rat brain. Neuroreport.

[128] Ting K.K., Brew B.J., Guillemin G.J. (2009). Effect of quinolinic acid on human astrocytes morphology and functions: Implications in Alzheimer’s disease. J. Neuroinflamm..

[129] Farooqui A.A., Yang H.C., Horrocks L. (1997). Involvement of phospholipase A2 in neurodegeneration. Neurochem. Int..

[130] Hardingham G.E. (2009). Coupling of the NMDA receptor to neuroprotective and neurodestructive events. Biochem. Soc. Trans..

[131] Sekine A., Okamoto M., Kanatani Y., Sano M., Shibata K., Fukuwatari T. (2015). Amino acids inhibit kynurenic acid formation via suppression of kynurenine uptake or kynurenic acid synthesis in rat brain in vitro. Springerplus.

[132] Braidy N., Grant R., Adams S., Brew B.J., Guillemin G.J. (2009). Mechanism for Quinolinic Acid Cytotoxicity in Human Astrocytes and Neurons. Neurotox. Res..

[133] Cammer W. (2001). Oligodendrocyte killing by quinolinic acid in vitro. Brain Res..

[134] Cammer W. (2002). Protection of cultured oligodendrocytes against tumor necrosis factor-α by the antioxidants coenzyme Q10 and N-acetyl cysteine. Brain Res. Bull..

[135] Chen Y., Brew B.J., Guillemin G.J. (2010). Characterization of the kynurenine pathway in NSC-34 cell line: Implications for amyotrophic lateral sclerosis. J. Neurochem..

[136] Vécsei L., Szalárdy L., Fülöp F., Toldi J. (2012). Kynurenines in the CNS: Recent advances and new questions. Nat. Rev. Drug Discov..

[137] Lim C.K., Bilgin A., Lovejoy D.B., Tan V., Bustamante S., Taylor B.V., Bessede A., Brew B.J., Guillemin G.J. (2017). Kynurenine pathway metabolomics predicts and provides mechanistic insight into multiple sclerosis progression. Sci. Rep..

[138] Rajda C., Galla Z., Polyák H., Maróti Z., Babarczy K., Pukoli D., Vécsei L. (2020). Cerebrospinal Fluid Neurofilament Light Chain Is Associated with Kynurenine Pathway Metabolite Changes in Multiple Sclerosis. Int. J. Mol. Sci..

[139] Saraste M., Matilainen M., Rajda C., Galla Z., Sucksdorff M., Vécsei L., Airas L. (2022). Association between microglial activation and serum kynurenine pathway metabolites in multiple sclerosis patients. Mult. Scler. Relat. Disord..

[140] Szabo M., Lajkó N., Dulka K., Barczánfalvi G., Lőrinczi B., Szatmári I., Mihály A., Vécsei L., Gulya K. (2023). The kynurenic acid analog SZR104 induces cytomorphological changes associated with the anti-inflammatory phenotype in cultured microglia. Sci. Rep..

[141] Szabo M., Lajkó N., Dulka K., Szatmári I., Fülöp F., Mihály A., Vécsei L., Gulya K. (2022). Kynurenic Acid and Its Analog SZR104 Exhibit Strong Antiinflammatory Effects and Alter the Intracellular Distribution and Methylation Patterns of H3 Histones in Immunochallenged Microglia-Enriched Cultures of Newborn Rat Brains. Int. J. Mol. Sci..

[142] Tai Y.-H., Engels D., Locatelli G., Emmanouilidis I., Fecher C., Theodorou D., Müller S.A., Licht-Mayer S., Kreutzfeldt M., Wagner I. (2023). Targeting the TCA cycle can ameliorate widespread axonal energy deficiency in neuroinflammatory lesions. Nat. Metab..

[143] Simkins T.J., Duncan G.J., Bourdette D. (2021). Chronic Demyelination and Axonal Degeneration in Multiple Sclerosis: Pathogenesis and Therapeutic Implications. Curr. Neurol. Neurosci. Rep..

[144] Waxman S.G. (2006). Axonal conduction and injury in multiple sclerosis: The role of sodium channels. Nat. Rev. Neurosci..

[145] Nikić I., Merkler D., Sorbara C., Brinkoetter M., Kreutzfeldt M., Bareyre F.M., Brück W., Bishop D., Misgeld T., Kerschensteiner M. (2011). A reversible form of axon damage in experimental autoimmune encephalomyelitis and multiple sclerosis. Nat. Med..

[146] Campbell G.R., Ziabreva I., Reeve A.K., Krishnan K.J., Reynolds R., Howell O., Lassmann H., Turnbull D.M., Mahad D.J. (2011). Mitochondrial DNA deletions and neurodegeneration in multiple sclerosis. Ann. Neurol..

[147] Ziabreva I., Campbell G., Rist J., Zambonin J., Rorbach J., Wydro M.M., Lassmann H., Franklin R.J.M., Mahad D. (2010). Injury and differentiation following inhibition of mitochondrial respiratory chain complex IV in rat oligodendrocytes. Glia.

[148] Giorgio A., De Stefano N. (2016). Advanced Structural and Functional Brain MRI in Multiple Sclerosis. Semin. Neurol..

[149] Garza-Lombó C., Posadas Y., Quintanar L., Gonsebatt M.E., Franco R. (2018). Neurotoxicity Linked to Dysfunctional Metal Ion Homeostasis and Xenobiotic Metal Exposure: Redox Signaling and Oxidative Stress. Antioxid. Redox Signal..

[150] Kwakye G.F., Paoliello M.M., Mukhopadhyay S., Bowman A.B., Aschner M. (2015). Manganese-Induced Parkinsonism and Parkinson’s Disease: Shared and Distinguishable Features. Int. J. Environ. Res. Public Health.

[151] Chtourou Y., Trabelsi K., Fetoui H., Mkannez G., Kallel H., Zeghal N. (2011). Manganese Induces Oxidative Stress, Redox State Unbalance and Disrupts Membrane Bound ATPases on Murine Neuroblastoma Cells In Vitro: Protective Role of Silymarin. Neurochem. Res..

[152] Molina-Holgado F., Hider R.C., Gaeta A., Williams R., Francis P. (2007). Metals ions and neurodegeneration. Biometals Int. J. Role Met. Ions Biol. Biochem. Med..

[153] Sayre L.M., Smith M.A., Perry G. (2001). Chemistry and Biochemistry of Oxidative Stress in Neurodegenerative Disease. Curr. Med. Chem..

[154] Bénardais K., Kotsiari A., Škuljec J., Koutsoudaki P.N., Gudi V., Singh V., Vulinović F., Skripuletz T., Stangel M. (2013). Cuprizone [Bis(Cyclohexylidenehydrazide)] is Selectively Toxic for Mature Oligodendrocytes. Neurotox. Res..

[155] Rossi-George A., Guo C.-J., Oakes B.L., Gow A.J. (2012). Copper modulates the phenotypic response of activated BV2 microglia through the release of nitric oxide. Nitric Oxide.

[156] Mahad D.H., Trapp B.D., Lassmann H. (2015). Pathological mechanisms in progressive multiple sclerosis. Lancet Neurol..

[157] Khalil M., Teunissen C., Langkammer C. (2011). Iron and Neurodegeneration in Multiple Sclerosis. Mult. Scler. Int..

[158] Lehmann I., Sack U., Lehmann J. (2011). Metal ions affecting the immune system. Met. Ions Life Sci..

[159] Pihl-Jensen G., Tsakiri A., Frederiksen J.L. (2015). Statin Treatment in Multiple Sclerosis: A Systematic Review and Meta-Analysis. CNS Drugs.

[160] Schoeps V.A., Graves J.S., Stern W.A., Zhang L., Nourbakhsh B., Mowry E.M., Henry R.G., Waubant E. (2022). N-Acetyl Cysteine as a Neuroprotective Agent in Progressive Multiple Sclerosis (NACPMS) trial: Study protocol for a randomized, double-blind, placebo-controlled add-on phase 2 trial. Contemp. Clin. Trials.

[161] Fitzgerald K.C., Morris B., Soroosh A., Balshi A., Maher D., Kaplin A., Nourbakhsh B. (2021). Pilot randomized active-placebo-controlled trial of low-dose ketamine for the treatment of multiple sclerosis-related fatigue. Mult. Scler..

[162] Green A.J., Gelfand J.M., Cree B.A., Bevan C., Boscardin W.J., Mei F., Inman J., Arnow S., Devereux M., Abounasr A. (2017). Clemastine fumarate as a remyelinating therapy for multiple sclerosis (ReBUILD): A randomised, controlled, double-blind, crossover trial. Lancet.

[163] Metz L.M., Li D., Traboulsee A., Myles M.L., Duquette P., Godin J., Constantin M., Yong V.W. (2009). Glatiramer acetate in combination with minocycline in patients with relapsing--remitting multiple sclerosis: Results of a Canadian, multicenter, double-blind, placebo-controlled trial. Mult. Scler..

[164] Metz L.M., Li D.K.B., Traboulsee A.L., Duquette P., Eliasziw M., Cerchiaro G., Greenfield J., Riddehough A., Yeung M., Kremenchutzky M. (2017). Trial of Minocycline in a Clinically Isolated Syndrome of Multiple Sclerosis. N. Engl. J. Med..

[165] Fox R.J., Coffey C.S., Conwit R., Cudkowicz M.E., Gleason T., Goodman A., Klawiter E.C., Matsuda K., McGovern M., Naismith R.T. (2018). Phase 2 Trial of Ibudilast in Progressive Multiple Sclerosis. N. Engl. J. Med..

[166] Montalban X., Arnold D.L., Weber M.S., Staikov I., Piasecka-Stryczynska K., Willmer J., Martin E.C., Dangond F., Syed S., Wolinsky J.S. (2019). Placebo-Controlled Trial of an Oral BTK Inhibitor in Multiple Sclerosis. N. Engl. J. Med..

[167] Sicotte N.L., Giesser B.S., Tandon V., Klutch R., Steiner B., Drain A.E., Shattuck D.W., Hull L., Wang H.J., Elashoff R.M. (2007). Testosterone treatment in multiple sclerosis: A pilot study. Arch. Neurol..

[168] Rajendran R., Böttiger G., Dentzien N., Rajendran V., Sharifi B., Ergün S., Stadelmann C., Karnati S., Berghoff M. (2021). Effects of FGFR Tyrosine Kinase Inhibition in OLN-93 Oligodendrocytes. Cells.

[169] Lagrèze W.A., Küchlin S., Ihorst G., Grotejohann B., Beisse F., Volkmann M., Albrecht P., Ungewiss J., Wörner M., Wolf S. (2021). Safety and efficacy of erythropoietin for the treatment of patients with optic neuritis (TONE): A randomised, double-blind, multicentre, placebo-controlled study. Lancet Neurol..

[170] Shingo T., Sorokan S.T., Shimazaki T., Weiss S. (2001). Erythropoietin regulates the in vitro and in vivo production of neuronal pro-genitors by mammalian forebrain neural stem cells. J. Neurosci. Off. J. Soc. Neurosci..

[171] Sanoobar M., Eghtesadi S., Azimi A., Khalili M., Khodadadi B., Jazayeri S., Gohari M.R., Aryaeian N. (2014). Coenzyme Q10 supplementation ameliorates inflammatory markers in patients with multiple sclerosis: A double blind, placebo, controlled randomized clinical trial. Nutr. Neurosci..

[172] Fiebiger S.M., Bros H., Grobosch T., Janssen A., Chanvillard C., Paul F., Dörr J., Millward J.M., Infante-Duarte C. (2013). The antioxidant idebenone fails to prevent or attenuate chronic experimental autoimmune encephalomyelitis in the mouse. J. Neuroimmunol..

[173] Mao P., Manczak M., Shirendeb U.P., Reddy P.H. (2013). MitoQ, a mitochondria-targeted antioxidant, delays disease progression and alleviates pathogenesis in an experimental autoimmune encephalomyelitis mouse model of multiple sclerosis. Biochim. Biophys. Acta (BBA)-Mol. Basis Dis..

[174] Feinstein D.L., Galea E., Gavrilyuk V., Brosnan C.F., Whitacre C.C., Dumitrescu-Ozimek L., Landreth G.E., Pershadsingh H.A., Weinberg G., Heneka M.T. (2002). Peroxisome proliferator-activated receptor-gamma agonists prevent experimental autoimmune encephalomyelitis. Ann. Neurol..

[175] Kantarci O.H., Lebrun C., Siva A., Keegan M.B., Azevedo C.J., Inglese M., Tintoré M., Newton B.D., Durand-Dubief F., Amato M.P. (2015). Primary Progressive Multiple Sclerosis Evolving from Radiologically Isolated Syndrome. Ann. Neurol..

